# Innovative probiotic fermentation approach for zearalenone detoxification in dried distiller’s grains

**DOI:** 10.3389/fmicb.2025.1533515

**Published:** 2025-06-02

**Authors:** Bilal Murtaza, Ling-ling Guo, Lili Wang, Xiaoyu Li, Liaqat Zeb, Bowen Jin, Ji-bin Li, Yongping Xu

**Affiliations:** ^1^MOE Key Laboratory of Bio-Intelligent Manufacturing, School of Bioengineering, Dalian University of Technology, Dalian, Liaoning, China; ^2^Dalian SEM Bioengineering Technology Co., Ltd., Dalian, China; ^3^Center for Food Safety of Animal Origin, Ministry of Education, Dalian University of Technology, Dalian, China; ^4^Microbial Research Institute of Liaoning Province, Chaoyang, China; ^5^Department of Chemistry, University of Bergen, Bergen, Norway

**Keywords:** feed safety, zearalenone mitigation, probiotic fermentation, DDGS, fungal toxin

## Abstract

Zearalenone (ZEN) contamination in dried distiller’s grains and solubles (DDGS) poses serious health risks and economic losses in animal farming. This study aimed to evaluate the effectiveness of probiotic fermentation using *Lactobacillus plantarum* CN1 in detoxifying ZEN and optimizing fermentation conditions for maximum efficiency. *L. plantarum* CN1, identified with 99% genetic homology, was used for DDGS fermentation. The detoxification mechanism was analyzed through adsorption assays, post-heat treatment effects, and scanning electron microscopy (SEM). ZEN removal was assessed over 72 h under various conditions, including bacterial concentration, temperature, and pH optimization. The results showed that CN1 achieved a maximum ZEN removal rate of 69% within 72 h, with an optimized efficiency of 75.6% at 4 × 10^9^ CFU/mL. Over 60% of ZEN was adsorbed by the bacterial cell wall, while removal in the fermentation supernatant and intracellular fluid remained below 5%. Scanning electron microscopy (SEM) analysis highlighted structural changes in the bacterial cells, particularly elongation and thinning, with more pronounced cell damage observed following heat and ZEN treatment. These modifications may explain the varying adsorption efficiencies observed. Heat treatment, particularly autoclaving, significantly enhanced adsorption efficiency to 82.9%, whereas acid and alkali treatments reduced it. Fermentation also improved the nutritional quality of DDGS, increasing crude protein by 7.16%, reducing crude fiber by 0.65%, and lowering pH to 4.3. These findings demonstrate that probiotic fermentation with CN1 offers a promising, cost-effective strategy for mitigating ZEN contamination while enhancing DDGS quality. Future studies should explore large-scale applications and the potential of CN1 in multi-mycotoxin detoxification to further improve feed safety.

## Graphical abstract

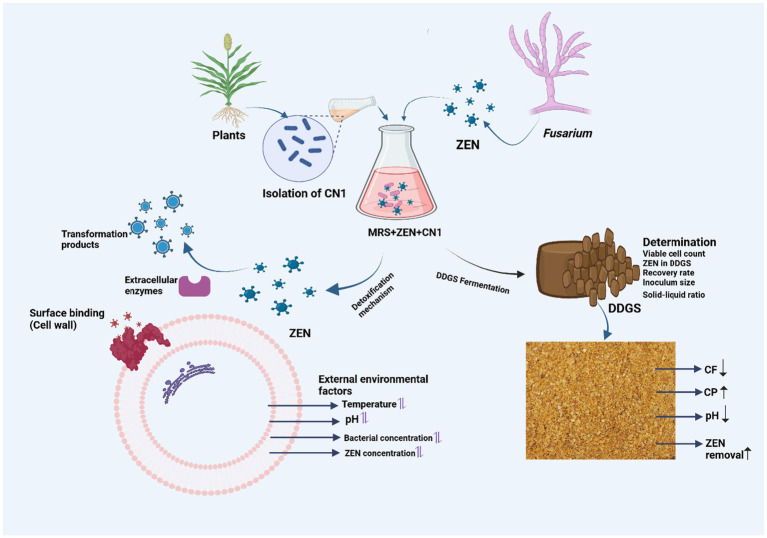

## 1 Introduction

Distiller’s dried grains with solubles (DDGS) is a high-quality protein feed derived from the fuel ethanol industry, which has grown significantly in recent years (Pont et al., 2023). The production of corn DDGS has increased, but it often contains high levels of mycotoxins, posing serious health risks. Mycotoxins are toxic secondary metabolites produced by fungi such as *Aspergillus*, *Penicillium*, and *Fusarium* under specific environmental conditions (Mohammadi Shad et al., 2021). Approximately 700 mycotoxins have been identified, with around 30 posing significant health threats to humans and animals, including carcinogenicity, immunotoxicity, and intestinal toxicity (Murtaza et al., 2024d; Sulyok et al., 2024).

Zearalenone (ZEN) is primarily produced by several species of *Fusarium*, which are known to contaminate cereals and other agricultural products (Murtaza et al., 2025). ZEN has a structure similar to 17-*β*-estradiol, giving it estrogenic properties that cause reproductive issues. ZEN binds to cytosolic estrogenic receptors in the uterus and mammary glands, leading to adverse effects such as pig ovarian atrophy, infertility, and abortion (Yang et al., 2021; Murtaza et al., 2022). Additionally, ZEN causes reproductive and cellular deformities, including atypical ovaries, abnormally shaped rat sperm, hypertrophied ovaries in sows, faulty embryos in zebrafish, and atrophy of seminiferous tubules (Murtaza et al., 2024a; Zhu et al., 2021). Because of these possible risks, ZEN contamination in food and animal feed must be addressed (Cai et al., 2022).

As a result of the danger that ZEN pollution poses, a great deal of study has gone into developing chemical, physical, and biological detoxification procedures. There are several physical and chemical techniques for removing ZEN, some of which include extrusion, grinding, washing, the adsorption of ozone, and hydrogen peroxide treatments (Wang et al., 2022; Ding et al., 2024). But there are certain drawbacks to these methods as well, such as significant nutrient losses and costly equipment and maintenance costs (Wang Y. et al., 2024). Because of its greater efficacy, selectivity, and little environmental impact, the biological detoxification strategy is strongly advised. Diverse bacterial and fungal species, such as *Bacillus* species (Wang et al., 2018b; Shi et al., 2024), *Lactobacillus plantarum* strain (Adunphatcharaphon et al., 2021; Murtaza et al., 2022), and *Rhizopus arrhizus* (Ahmed et al., 2024) have exhibited the capability for ZEN degradation. However, it is imperative to acknowledge that ZEN degradation may not culminate in complete detoxification, potentially giving rise to more estrogenic derivatives, including *α*-zearalenol (α-ZAL) and *β*-zearalenol (β-ZAL; Murtaza et al., 2023b, 2024b). Therefore, achieving comprehensive ZEN detoxification requires a multifaceted process involving multiple enzymes and new strains.

Thus, *Lactobacillus*, which is renowned for its resilience, was evaluated for its potential to lower ZEN levels in order to get a thorough understanding of ZEN elimination in different *Lactobacillus* species. This study aimed to address the critical gap in identifying effective probiotic strains capable of detoxifying ZEN in DDGS, a challenge that poses significant health risks and economic losses in animal feed production. Current methods for ZEN detoxification are often inefficient or costly, highlighting the need for innovative biological approaches. To bridge this gap, we focused on isolating and characterizing a new ZEN-removing probiotic strain, *Lactobacillus plantarum* CN1. Our *in vitro* research assessed the detoxification capacity of this strain under various conditions, including different ZEN concentrations, incubation pH, temperatures, and the influence of the bacterial cell wall. We specifically aimed to evaluate the adsorption mechanisms of CN1, optimize the fermentation medium, and explore the combined use of probiotics to enhance the nutritional value of DDGS while reducing its mycotoxin content.

## 2 Materials and methods

### 2.1 Chemicals and culture media

Zearalenone (ZEN) standard and De Man, Rogosa, and Sharpe agar (MRS) from Merck (Darmstadt, Germany) were used to culture the plant-derived bacteria. Chemicals such as NaCl, KCl, Na_2_HPO_4_, and KH_2_PO_4_ were supplied by Carlo Erba Reagents (Bangkok, Thailand). ZEN (99%) was obtained from TRC (Canada). ZEN detection kits were provided by Heilongjiang Wanlird Biotechnology Co., Ltd., and Qingdao Pribolab Biological Engineering Co., Ltd. DDGS was purchased from Heilongjiang Wanli Runda Biotechnology Co., Ltd. Other chemicals used included hydrochloric acid from Beijing Chemical Plant and sodium hydroxide from Shanghai McLean Biochemical Technology Co., Ltd.

MRS medium: MRS medium was prepared by dissolving the appropriate amount of MRS powder in distilled water, as per the manufacturer’s instructions. The mixture was heated and stirred until fully dissolved. The pH was adjusted to 6.2–6.6 using a pH meter, and the medium was sterilized by autoclaving at 121°C for 15 min. After sterilization, the medium was allowed to cool to room temperature before use.

Inorganic salt medium (MM): Inorganic salt medium (MM) was prepared by dissolving 1 g of (NH_4_)2SO_4_, 0.2 g of MgSO_4_, 0.1 g of CaCl_2_, 0.5 g of NaH_2_PO_4_, and 0.5 g of KH_2_PO_4_ in distilled water to a final volume of 1,000 mL. The pH was adjusted to 7.0, and the medium was autoclaved at 121°C for 25 min. For solid culture, 2% agar was added before sterilization. The preparation of this medium was based on the protocols described by Wang et al. (2018a).

### 2.2 Screening of anaerobic strains

Dissolve 1 g of each of the 18 purchased lactic acid bacteria products in 4 mL of PBS buffer and vortex for 1 min. Inoculate 1 mL of this suspension into 10 mL of MRS liquid culture medium and incubate anaerobically at 37°C for 24 h. Following incubation, dilute the culture 100-fold with PBS and spread it onto MRS medium plates. Place the plates in an anaerobic bag and incubate at 37°C for 24 h. Select single colonies of different shapes from the plates and streak them onto fresh MRS solid medium plates for further culturing. After multiple purification steps, when the colonies exhibit consistent growth patterns, select single colonies for preservation. For enrichment, inoculate the selected single colonies into 10 mL of MRS liquid medium and incubate anaerobically at 37°C for 24 h. Centrifuge the culture at 4°C at 7168 × g for 10 min, discard the supernatant, and resuspend the bacterial cells in MM liquid medium, adjusting the volume to 5 mL. Inoculate 0.5 mL (10% of the inoculum volume) into 0.5 mL of rescreening medium and incubate on a shaker at 37°C and 160 × g for 72 h. Adjust the volume to 1 mL with MM liquid culture medium, centrifuge at 9775 × g for 10 min, and filter the supernatant through a 0.22 μm filter membrane. Prepare samples for HPLC detection from the filtered supernatant.

### 2.3 Morphological observation and 16S rDNA sequencing

After the purification process, streaks of the chosen bacterial strains were applied onto MRS solid culture media plates. These plates underwent static incubation in a 37°C constant-temperature incubator for 12 and 24 h, respectively. Throughout the incubation period, close observation was carried out to record the characteristics of bacterial colonies, encompassing morphology, color, size, and texture. Single colonies demonstrating unique features were carefully chosen for subsequent analysis. Gram staining techniques were then utilized to categorize the bacterial strains according to their cell wall composition, followed by microscopic examination to evaluate cell morphology and arrangement.

Bacterial DNA was isolated using a simplified boiling method. Briefly, 1 mL of bacterial suspension, with concentrations ranging from 10^8^ to 10^0^ CFU/mL, was subjected to centrifugation at 8000 × g for 2 min to pellet the cells. The supernatant was discarded, and the pellet was washed by resuspension in sterile deionized water, followed by a second centrifugation step under the same conditions. The resulting pellet was then resuspended in 100 μL of sterile deionized water and incubated at 100°C for 10 min to facilitate cell lysis. After heat treatment, the suspension was centrifuged again at 8000 × g for 2 min, and the resulting supernatant, containing crude genomic DNA, was used as the template for subsequent analysis (Wang et al., 2020). The targeted gene amplified via PCR is the 16S rRNA gene, utilizing the universal primer pair: 27F (5’-AGAGTTTGATCMTGGCTCAG-3′) and 1492R (5’-GGTTACCTTGTTACGACTT-3′). The PCR amplification program comprises a pre-denaturation step at 94°C for 5 min, followed by 35 cycles of denaturation at 94°C for 30 S, annealing at 55°C for 30 S, and extension at 72°C for 90 S. Finally, a single extension step is carried out at 72°C for 10 min. Subsequently, the resultant PCR products are sent to Shanghai, Sangon, for sequencing. The sequencing outcomes are then compared through Blast in NCBI to analyze their homology. Using MEGA 11 software, the identified homologous sequences are aligned with the target sequences, employing the neighbor-joining method for sequence alignment and homology analysis. Finally, a phylogenetic tree is constructed to elucidate the evolutionary relationships among the examined bacterial strains.

### 2.4 High-performance liquid chromatography and determination of adsorption percentage (ADS%)

The HPLC analysis of ZEN-containing materials was conducted following the method described by Kim and Vujanovic (2017). The centrifuged supernatant (0.5 mL) was diluted with HPLC-grade methanol, processed for 20 min, and filtered through a 0.22 μm organic membrane filter before analysis. ZEN levels were quantified using an Agilent 1,260 Infinity II HPLC system (Agilent Technologies, China) equipped with a fluorescence detector (FLD) and an Agilent ZORBAX Eclipse Plus C18 column (4.6 mm × 250 mm, 5 μm). The operational column temperature was 30°C, with a mobile phase composition of acetonitrile/water (40:60, v/v) and a flow rate of 1.0 mL/min. The sample injection volume was 15 μL, and detection was carried out at an excitation wavelength of 236 nm and an emission wavelength of 450 nm. The limit of detection (LOD) and limit of quantification (LOQ) were determined as 0.02 ppm and 0.06 ppm, respectively. Calibration was performed using a standard ZEN solution prepared in methanol, with a five-point calibration curve (R^2^ > 0.999) ensuring accuracy. The adsorption amount was determined by comparing the peak areas of ZEN in the post-adsorption supernatant to those in the control solution. The ZEN adsorption efficiency (ADS%) was calculated using Equation 1 (Wei et al., 2019).

As expressed in the following formula:


(1) ADS=100−AUC1/AUC2×100.

Where:

*ADS* represents the adsorption percentage.

*AUC2* represents the area where the ZEN peaks in the supernatant.

*AUC1* is used to describe the region that contains the ZEN peak in the control positive.

### 2.5 ZEN removal and adsorption by CN1 strain

The CN1 bacteria were initially cultured on MRS agar for 48 h at 37°C. Individual colonies were then transferred into 5 mL of MRS culture medium containing ZEN at a concentration of 10 μg/mL and incubated for 72 h at 37°C. The ZEN-degrading strain was subsequently preserved in glycerol (25%) and stored at-80°C until further use. For the experiment, the strain was inoculated into 30 mL of MRS broth and incubated in a shaker incubator (ZWY-211B, LABWIT Scientific Pty. Ltd., Australia) at 37°C and 150 × g for 48 h. Following incubation, the cells and supernatants were separated via centrifugation at 7168 × g for 20 min at 4°C. The supernatants were then analyzed for residual ZEN content. Additionally, isolated cells were disrupted using an ultrasonic disruptor after resuspension in PBS. The disrupted cells were centrifuged, the supernatant was discarded, and the resulting broken residue (bacterial cell wall) was preserved. Subsequently, the inactivated bacteria solution and broken residue were incubated with 10 μg/mL ZEN for 72 h at 37°C and 150 × g, and ZEN levels were detected using HPLC.

### 2.6 ZEN removal by CN1 strain post-acid and heat treatments

After culturing CN1 cells at 37°C, the cells were extracted and mixed with 2 M HCl and phosphate-buffered saline (PBS, pH 7.0, 1 M) for 1 h. The final pH of the acid-treated suspension reached approximately pH 1.5, while the alkali-treated samples reached approximately pH 12.5. Subsequently, each pellet was autoclaved at 121°C for 15 min. The cells were then centrifuged at 5488 × g for 5 min to remove them. To achieve the desired concentration, bacteria at 4 × 10^9^ (CFU/mL) were suspended in 5 mL of PBS (pH 7.0). A 1 mL sample was taken from this suspension and centrifuged at 5488 × g for 8 min at 4°C before being analyzed using HPLC.

### 2.7 ZEN removal by CN1 under varying conditions

After cultivating CN1 cells, a concentration of 4 × 10^9^ CFU/mL of bacterial cells was added to 5 mL of MRS broth with a pH of 7.0, along with varying concentrations of ZEN (2.5, 5, 8.5, and 200 μg/mL). This mixture was then incubated at 37°C for 72 h with agitation at 150 × g. After incubation, 1 mL samples were taken and centrifuged at 5488 × g for 10 min at 4°C. CN1 cells were also introduced into 5 mL of MRS broth with varying pH levels (3.0, 6.0, 7.0, and 8.0) containing 10 μg/mL ZEN to reach a concentration of 4 × 10^9^ CFU/mL. These samples were incubated for 72 h at 37°C with agitation at 150 × g. ZEN levels were measured using HPLC after centrifuging 1 mL samples at 5488 × g for 10 min at 4°C. Before testing, the MRS broth was brought to temperatures of 4, 25, 37, and 42°C. CN1 cell density reached 4 × 10^9^ CFU/mL in 5 mL of LB broth containing 10 μg/mL ZEN. Each sample was agitated for 72 h at 4, 25, 37, and 42°C with agitation at 150 × g. After incubation, toxin concentrations were determined using HPLC following centrifugation of 1 mL samples at 5488 × g for 10 min at 4°C. After 72 h of cultivation, CN1 cells were introduced into 10 mL of MRS broth with a pH of 7.0 containing 10 μg/mL of ZEN and different bacterial concentrations (4 × 10^9^, 2 × 10^9^, 4 × 10^8^, and 2 × 10^8^ CFU/mL). These mixtures were continuously agitated for 3 d at 150 × g and 37°C. Following centrifugation of the samples at 5488 × g for 10 min at 4°C, the concentrations of ZEN were assessed using HPLC. In a shaker operating at 37°C and 150 × g for 48 h, CN1 cells were added to 30 mL of MRS broth. After incubation, the bacterial cells were separated from the culture supernatant by centrifugation at 5488 × g for 8 min at 4°C. The isolated cells in PBS were then disrupted using ultrasonic waves, and the resulting mixture was centrifuged again to separate the bacterial cell wall residue. The ZEN concentration in the culture supernatant, disrupted supernatant, and residual cell wall was maintained at 10 μg/mL. ZEN levels were measured using HPLC after 72 h in the shaker at 37°C and 150 × g.

### 2.8 Scanning electron microscopy analysis

The Scanning Electron Microscope (SEM) analysis was conducted to investigate the morphological changes in bacterial samples, specifically strain CN1 treated with ZEN and heat. The analysis was performed using a NOVA NANOSEM-450 scanning electron microscope (FEI, United States). The bacterial pellets, subjected to different treatments, were fixed using a modified Karnofsky’s solution containing 1.6% glutaraldehyde and 2.6% paraformaldehyde in a 0.1 M sodium phosphate buffer (pH 7.2). After a 24-h incubation at 4°C, the pellets were dehydrated through a series of ethyl alcohol and acetone solutions with 10-min intervals between each concentration. The ethyl alcohol concentrations used were 30, 50, 70, 90, and 100%, with 10-min breaks between each stage. Subsequently, the samples were dried, gold-coated using an ion sputter JFC-1100 (JEOL, Japan), and secured to stubs using carbon tape. SEM images were acquired to examine bacterial morphology and elemental composition (Ge et al., 2017).

### 2.9 Determination of strain tolerance to ZEN

The CN1 strain was cultured and enriched, adjusting bacterial concentrations to 1 × 10^9^ CFU/mL before inoculating them into MRS culture media with a 1% inoculation volume and a 1 mL culture system. Each reaction space in a centrifuge tube was 1.5 mL, and various ZEN concentrations from 0 to 100 μg/mL were prepared. After thorough mixing, 200 μL of each sample was dispensed into a 96-well plate and incubated in a microplate reader at 37°C with shaking for 18 h. Measurements were taken every hour at a wavelength of 600 nm, allowing for the continuous monitoring of bacterial growth and activity in response to different ZEN concentrations.

### 2.10 Effects of different treatment methods on ZEN adsorption by CN1 strain

To assess the potential of CN1 cells for ZEN removal via cell wall adsorption, the cells were deactivated using various methods. Following deactivation, the cells were collected through centrifugation, washed twice with PBS, and adjusted to a bacterial concentration of 1 × 10^10^ CFU/mL. The washed cells were divided into five groups for different treatments: A. Autoclaving: Cells were autoclaved at 121°C for 20 min. B. Water bath: Cells were subjected to a 100°C water bath for 40 min. C. Acid treatment: Cells were treated with 2 mL of 1 M HCl at 37°C with shaking at 160 × g for 1 h. D. Alkali treatment: Cells were treated with 2 mL of 1 M NaOH at 37°C with shaking at 160 × g for 1 h. E. Control group: No treatment was administered to this group. The treated bacterial cells from each group were centrifuged at 5488 × g and 4°C for 10 min, followed by resuspension in 2 mL of MM liquid culture medium. ZEN was introduced into each suspension to achieve a concentration of 20 μg/mL. These suspensions were then placed on a shaker at 37°C with agitation at 160 × g for 72 h. After the incubation period, the suspensions underwent centrifugation at 5488 × g for 10 min at 4°C, and the resulting supernatant was filtered through a 0.22 μm filter to prepare the samples. HPLC analysis was employed to detect the concentration of ZEN in the samples. A control group with a ZEN solution of the same concentration but without added bacteria was included for comparison, and each group underwent triplicate testing to ensure statistical validity.

### 2.11 Adsorption stability of CN1 strain to ZEN

After the activation of CN1 cells, they were centrifuged at 4°C, 5488 × g for 10 min to gather them. Following that, the cells underwent two washes with PBS buffer to eliminate any potential interference from the culture medium. Next, the cells were suspended in an MM culture medium with a final concentration of 20 μg/mL ZEN. This suspension was then placed in a shaker at 160 × g and incubated at 37°C for 24 h. Following the incubation period, the suspension underwent centrifugation at 5488 × g for 10 min, and the resulting supernatant was sampled for testing. The pellets were resuspended with 1 mL of MM culture medium and divided into three groups for further treatment. Group A underwent incubation at 80°C for 10 min, group B was vortexed for 5 min, and group C was treated with 1 mL of 95% ethanol and incubated at 37°C for 10 min. Post-treatment, each group underwent centrifugation at 5488 × g for 10 min, and the supernatant of each group was collected. These collected supernatants were then passed through a 0.22 μm filter membrane and subjected to HPLC analysis to determine the desorption rate. The desorption rate (M) was calculated using the formula: M = (B/B0) × 100%, where B0 represents the adsorption amount of CN1 bacteria and B is the ZEN content in the supernatant after treatment.

### 2.12 Determination of nutritional components of fermented DDGS

#### 2.12.1 Fermentation culture process and crude protein determination

Under sterile conditions, transfer the fermentation product into a zip-lock bag with a one-way air valve and inoculate with 5% CN1. Seal the bag using a heating plastic sealing machine and incubate anaerobically for 14 d. Based on the findings in Section 2.7, maintain the fermentation temperature at 37°C and the initial pH at 6.5 for both strains. The determination of crude protein was performed following the guidelines outlined in the GB/T 6432–2018 standard, utilizing a Kjeldahl nitrogen analyzer. In this method, a 1.0 g sample was digested with a mixture of sulfuric acid, potassium sulfate, and copper sulfate. Subsequently, the nitrogen compounds were distilled, converting them to ammonium sulfate, which was then released as ammonia and absorbed in boric acid. The ammonia was titrated with hydrochloric acid using methyl red and bromocresol blue indicators. The crude protein content was calculated by multiplying the nitrogen content by 6.25. The digestion process involved boiling at 150°C for 30 min, followed by heating at 280°C for another 30 min, and finally at 380°C for 2.5 h. Each experiment was conducted in triplicate, including a blank control group for comparison.

#### 2.12.2 Crude fiber and pH determination

The determination of crude fiber followed the GB/T 6434–2006 standard protocol. Initially, the sample was weighed and subjected to digestion with acid and alkali solutions. Ether and acetone were then used to eliminate ether-soluble components. High-temperature burning was employed to remove minerals, leaving behind the crude fiber content for measurement. This analytical procedure was conducted at the Animal Nutrition and Feed Research Institute in Changchun. Specifically, 10 g of the sample were mixed with 100 mL of ultrapure water, stirred for 10 min with a magnetic stirrer, and filtered through gauze. The pH of the filtrate was measured using a pH meter as part of the process.

#### 2.12.3 ZEN determination by ELISA assay

The indirect competitive ELISA method involves pre-coating a ZEN antigen on an enzyme-labeled microplate. ELISA was selected as a practical and validated method suitable for rapid, high-throughput screening in complex feed matrices. The primary goal was to compare ZEN levels between treated and untreated DDGS samples, rather than to obtain absolute quantification. For sample preparation, 5 g of fermented DDGS are weighed into a 50 mL centrifuge tube and mixed with ultrapure water. After vortexing for 10 min, the sample is sonicated for 30 min and then centrifuged at 1792 × g. A 100 μL portion of the clear liquid is taken, mixed with 400 μL of ultrapure water, and prepared as the sample to be tested. To verify the recovery rate, the pre-fermentation sample is weighed and then spiked with standard ZEN solutions to achieve concentrations of 1 μg/mL, 5 μg/mL, and 10 μg/mL in the fermentation medium. Three replicates are done for each concentration without adding microbial agents. The same sample preparation steps are followed as in the fermentation process, and statistical methods are used to calculate the ZEN content and recovery rate in the samples.

### 2.13 Statistical analysis

The experiments were conducted with three technical replicates each. The mean standard error (X ± SE) for all statistical analyses was calculated using GraphPad Prism. Group mean differences were assessed using the one-way analysis of variance (ANOVA), with significance denoted by *p < 0.05*. In the DDGS with ZEN study, data processing and analysis were performed using Origin2023.

## 3 Results and discussion

### 3.1 Screening of anaerobic strains

**Figure 1:**
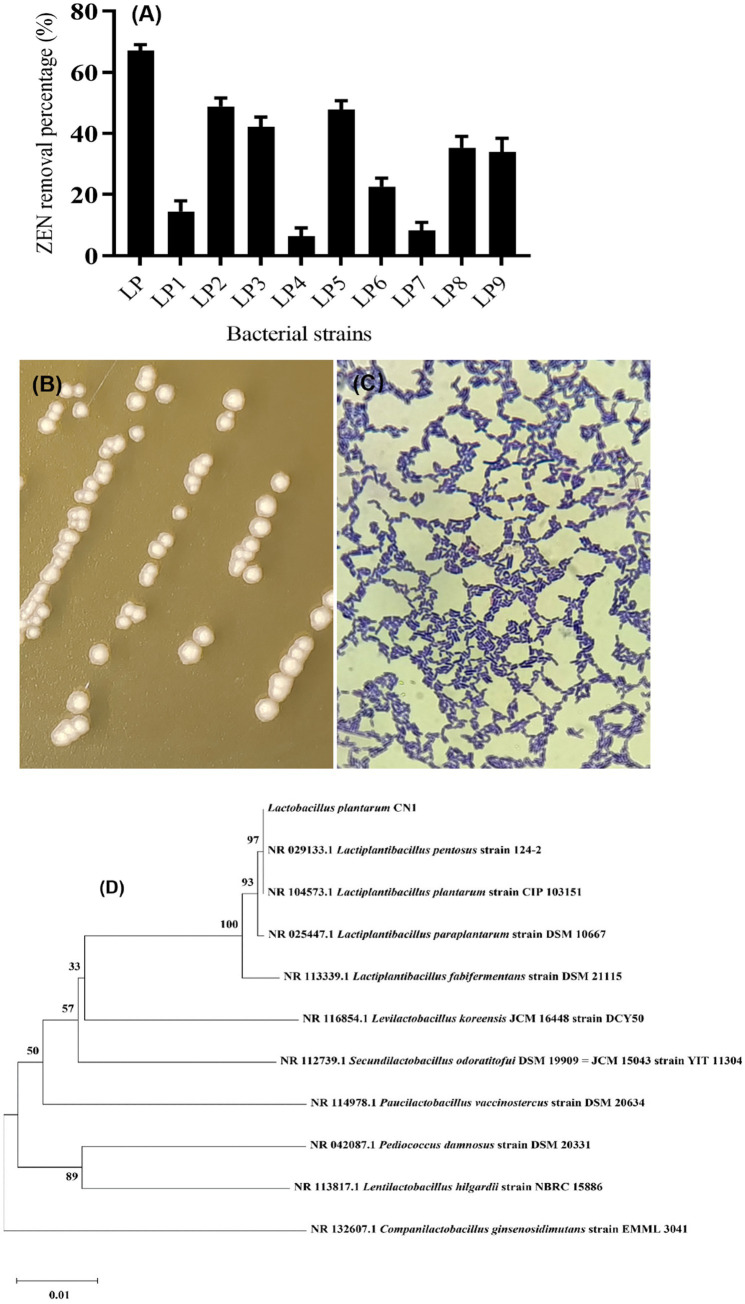
**(A)** Screening results of ZEN removal strains; **(B)** Colony morphology of CN1 strain; **(C)** Gram staining; **(D)** Phylogenetic tree constructed by gene sequence of strain CN1 16S rDNA.

The ZEN removal ability of 10 strains of lactic acid bacteria from different probiotic products was studied using a re-screening method. It was found that 5 strains had a significant effect on ZEN removal, as shown in Figure 1A. The strain with the highest removal ability, LP (CN1), achieved a 69% removal rate, while the strain with the lowest, LP4, had a removal rate of only 9.1%. Strains LP2, LP3, and LP5, with removal rates greater than 40%, were selected for further research. During the initial screening, none of the 16 lactic acid bacteria strains could grow on inorganic salt medium plates with ZEN as the sole carbon source. However, 5 strains demonstrated removal effects in MM medium liquid with ZEN as the sole carbon source. Current research indicates that most lactic acid bacteria cannot use ZEN for growth, and toxin removal is primarily due to the physical adsorption by the cell wall, reducing toxin concentration. The specific degradation mechanisms of ZEN by each strain will be studied in subsequent experiments.

### 3.2 Morphological and molecular characteristics of strains

The identification of the CN1 strain through colony characteristics, Gram staining (Figure 1C), and 16S rDNA sequencing is a standard approach that aligns with methods used in previous studies for microbial identification. The description of the CN1 strain colonies, with a milky white center, translucent edges, and smooth, moist surfaces with ridges, is consistent with typical descriptions of *Lactobacillus plantarum* colonies (Figure 1B). Ricciardi et al. (2015) described similar morphological characteristics in their identification of *Lactobacillus plantarum* strains isolated from fermented foods. The amplification of the 16S rDNA conserved sequence and subsequent phylogenetic analysis using tools like MEGA11 further validate the identification process. Kim and Vujanovic (2017) utilized 16S rDNA sequencing to accurately identify and classify *Lactobacillus* strains from various environments. The phylogenetic tree constructed to identify the LP strain as *Lactobacillus plantarum* CN1 provides robust support for its classification (Figure 1D). This approach is corroborated by the study of Seddik et al. (2017), who also employed phylogenetic analysis to distinguish between closely related *Lactobacillus* species, emphasizing the reliability of this method. According to the Ministry of Agriculture of the People’s Republic of China’s Announcement No. 2045 in the “Feed Additive Catalog (2013),” *Lactobacillus plantarum* CN1 is approved for use as a feed additive. This regulatory context ensures that the strain meets the safety and efficacy standards required for inclusion in animal feed.

### 3.3 ZEN degradation and adsorption capacity of CN1 strain

**Figure 2:**
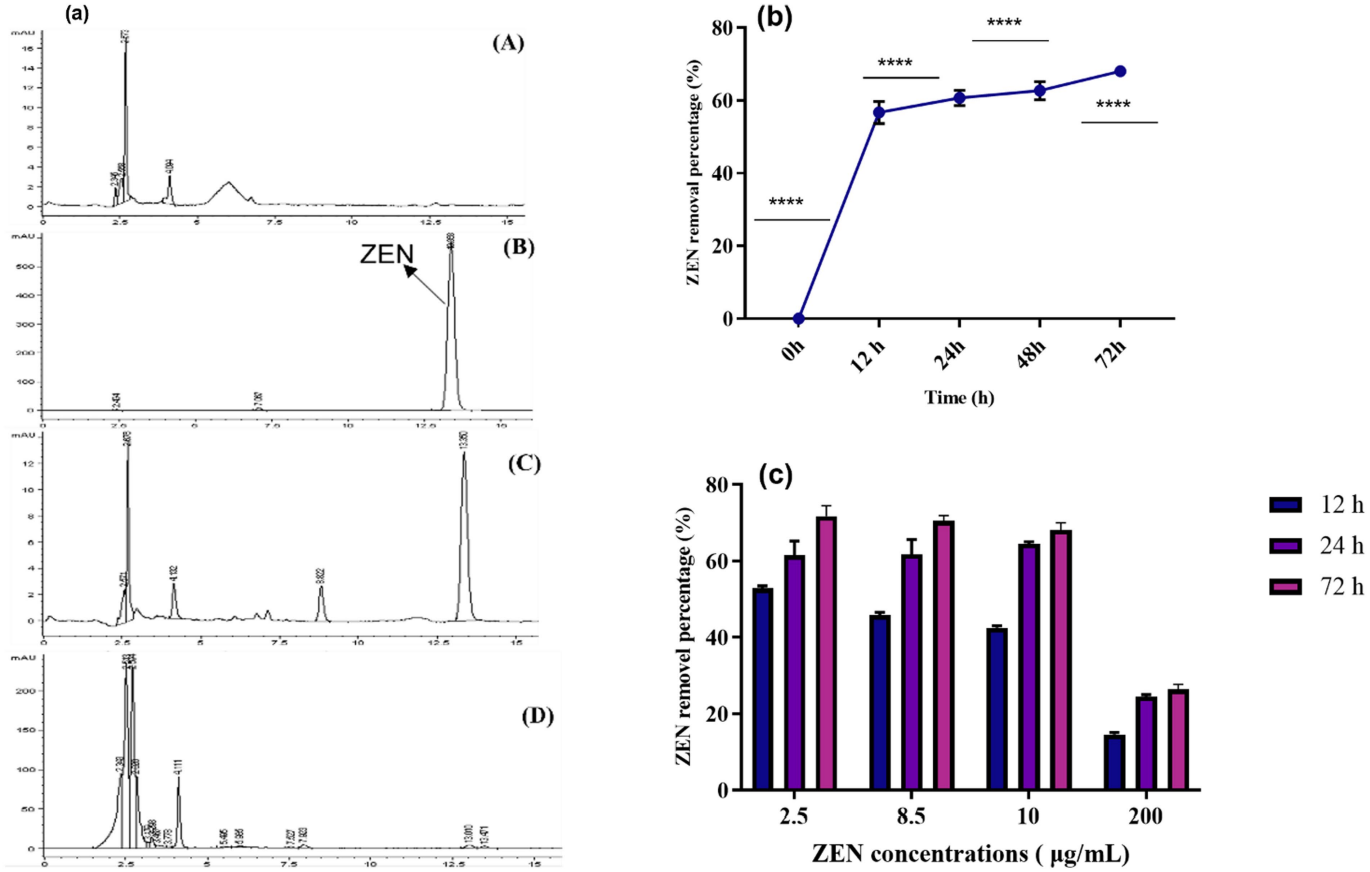
**(a)** HPLC of ZEN removal. ZEN (10 μg/mL) was added to CN1 and cultured in the MRS medium for 72 h, and the removal rate was then calculated using HPLC. (A) MRS medium; (B) Control group (ZEN toxin); (C) MRS medium containing ZEN toxin. (D) (ZEN in MRS medium+ CN1) MRS + ZEN with CN1 strain; **(b)** ZEN removal (%) was calculated; **(c)** ZEN removal ability of CN1 under acid and heat conditions. At 37°C for 72 h, CN1 cells (4 × 10^9^ CFU/mL) and MRS broth with various ZEN concentrations (2.5, 8.5, 10, and 200 μg/mL) were incubated. One-way ANOVA was used to analyze significant differences (*p < 0.05*).

This research is the first to investigate the mycotoxin-reducing potential of vegetative plant-derived *Lactobacillus* strains, which are utilized in a variety of fermented foods traditionally prepared throughout Asia and Southeast Asia. The ability of the CN1 strain to remove ZEN within 72 h was evaluated. Initially, the removal rate of ZEN by CN1 exceeded 60% within the first 12 h. This rate remained relatively stable between 12 and 72 h, ultimately reaching a maximum removal rate of 69% at 72 h (Figure 2b). Microorganisms employ processes such as ring cleavage, acetylation, deamination, and hydrolysis to biodegrade or transform mycotoxins (Hathout and Aly, 2014; Li et al., 2021; Murtaza et al., 2024c). The adsorption mechanism is likely governed by non-covalent interactions between ZEN hydrophobic backbone and functional groups present on the bacterial cell wall, such as peptidoglycan, surface-layer proteins, and teichoic acids. These components offer binding sites that facilitate hydrogen bonding and hydrophobic interactions, consistent with previous findings on lactic acid bacteria mycotoxin interactions (Wang X. et al., 2024). Bentonite and other clay-based adsorbents have shown 97% ZEA removal *in vitro* but often face limitations in specificity and nutrient binding (Ahn et al., 2022). Similarly, yeast cell walls, especially those enriched in *β*-glucans and mannoproteins, offer ZEA adsorption rates of around 68%, depending on the strain and processing conditions (Joannis-Cassan et al., 2011). In contrast, CN1 achieved a removal efficiency of 69% under optimal conditions in our study, which is comparable to or better than many of these agents. Previous research indicates that lactic acid bacteria, such as *Lactobacillus plantarum*, primarily degrade toxins through physical adsorption mechanisms (Vega et al., 2017). During the adsorption process, physical binding between the bacterial cell wall and the toxin can be somewhat unstable, leading to fluctuations in the adsorption rate. This instability might account for the observed variations in the removal efficiency over time. Pourmohammadi et al. (2022) demonstrated that the adsorption of mycotoxins by lactic acid bacteria could be influenced by environmental factors such as pH and temperature, which affect the stability of the adsorption process. The decline in the strain degradation ability over time might also be attributed to the natural lifecycle of the bacteria, where metabolic activity diminishes as the cells enter the stationary or decline phases (Rasmussen et al., 2015).

### 3.4 HPLC analysis of ZEN removal

In Figure 2a, the HPLC peaks illustrate the comparative analysis of groups A (MRS medium), B (control group), C (ZEN + MRS medium), and D (ZEN in MRS medium + CN1). The control group displayed a distinct peak at 13.3 min. In the ZEN + MRS medium group, two peaks were observed, indicating the presence of ZEN in the medium. Notably, the peak area was significantly reduced in the ZEN in the MRS medium + CN1 group, suggesting a decrease in ZEN concentration. These results demonstrate that after 72 h of incubation in MRS medium at 37°C, the CN1 strain effectively removed 69% of ZEN at a concentration of 10 μg/mL. This reduction is consistent with findings from other studies that highlight the capability of *Lactobacillus plantarum* strains to adsorb and degrade mycotoxins such as ZEN. El-Nezami et al. (2002) have documented similar adsorption and degradation capacities in lactic acid bacteria, emphasizing their potential use in detoxifying contaminated feeds.

### 3.5 ZEN removal by CN1 strain under acid and heat treatments

The influence of acid and heat treatment on the ZEN removal rate by CN1 (4 × 10^9^ CFU/mL) in MRS broth with ZEN concentrations ranging from 2.5 to 200 μg/mL was studied. CN1 achieved a removal rate of over 40% for ZEN at concentrations of 2.5 and 8.5 μg/mL after 12 h. Specifically, at ZEN concentrations of 10 and 200 μg/mL, CN1 removed 42.5 and 14.77% of ZEN, respectively. After 72 h, CN1 demonstrated higher ZEN removal efficiency at 2.5 and 8.5 μg/mL. Overall, CN1 significantly removed more ZEN at higher concentrations (2.5, 8.5, and 10 μg/mL) after 72 h (Figure 2c). Our results on CN1 resilience to HCl and heat treatment for ZEN removal align with findings on bacterial tolerance in acidic and thermal environments, highlighting potential challenges and adaptations. The application of strong acid and alkali treatments may alter the structural integrity of bacterial cell wall components, particularly peptidoglycan and surface proteins, potentially affecting ZEN adsorption behavior. These treatments were used to explore the contribution of surface components under extreme conditions, but may not reflect physiological interactions *in vivo*. Studies show that acidic environments, like gastric HCl, can impair bacterial cell membranes and enzymatic function, thus reducing ZEN degradation (Guan and Liu, 2020). Additionally, while moderate heat may stimulate some microbial activity, excessive heat can denature proteins critical for biodegradation (Laskowska et al., 1996). Consequently, to maintain CN1 efficacy in feed, future applications may consider delivery methods that shield the bacteria until they reach the intestines. These findings align with previous studies that underscore the effectiveness of lactic acid bacteria in mycotoxin removal. Previous studies demonstrated the ability of various *Lactobacillus* species to reduce aflatoxin levels through adsorption mechanisms (Li et al., 2021; Murtaza et al., 2024b). Additionally, Liu et al. (2019) highlighted that lactic acid bacteria can remove mycotoxins like ZEN through cell wall adsorption, similar to the findings in this study.

### 3.6 ZEN removal by CN1 strain at varying concentrations, pH levels, and temperatures

**Figure 3:**
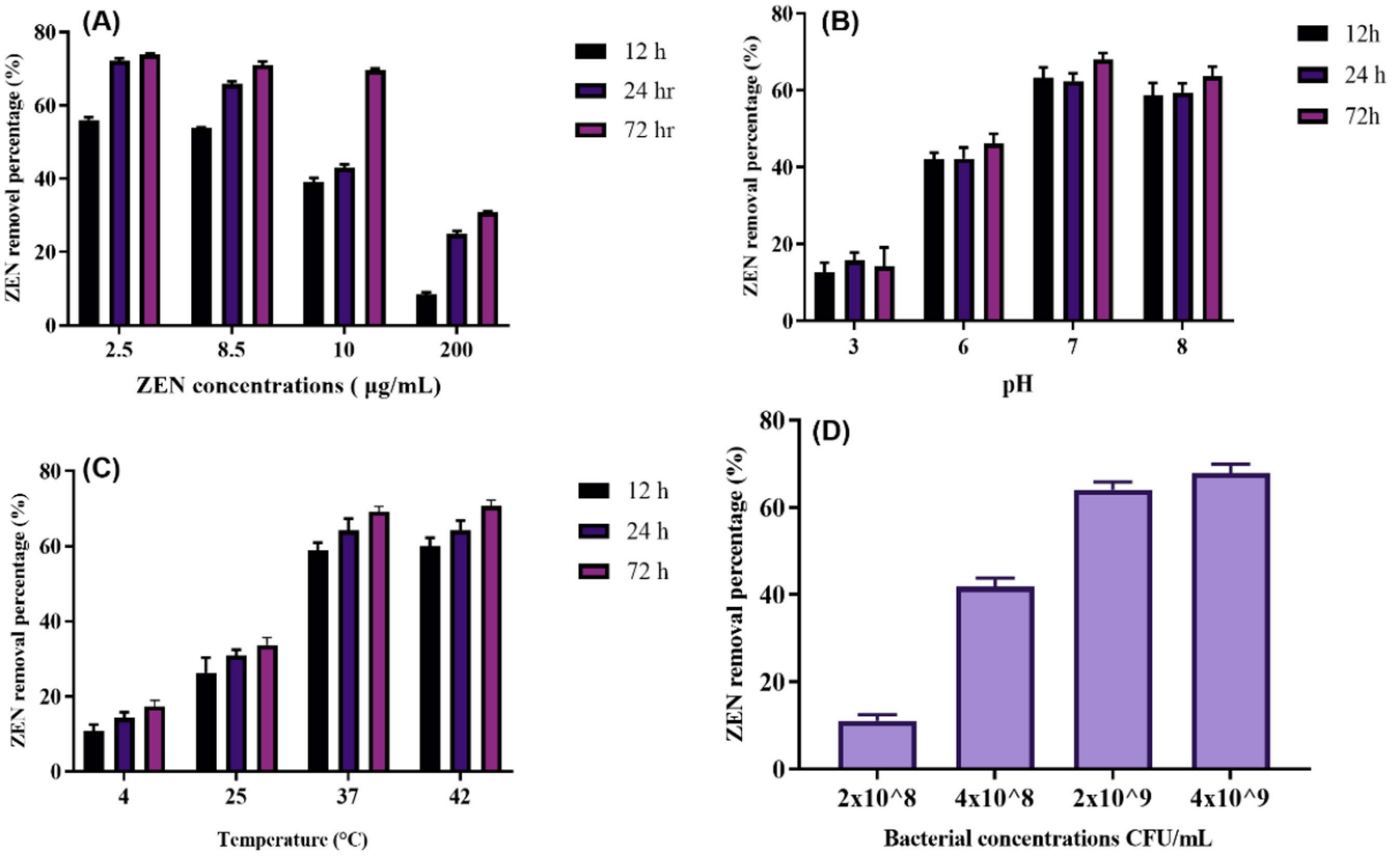
ZEN removing ability of CN1 under different ZEN-concentrations, pH, temperature and bacterial concentrations: **(A)** The 72-h incubation at 37°C with CN1 cells (4 × 10^9^ CFU/mL) in MRS broth with various concentrations of ZEN (2.5, 8.5, 10, and 200 μg/mL); **(B)** A final concentration of 4 × 10^9^ CFU/mL of bacterium cells were achieved by adding 5 mL of MRS broth (pH 3.0, 6.0, 7.0, and 8.0) with 10 μg/mL of ZEN to CN1 cells throughout a 72-h period at 37°C; **(C)** 5 mL of MRS broth containing 10 μg/mL of ZEN was added to the CN1 cells to create a final concentration of 4 × 10^9^ CFU/mL of bacterium cells. It was discovered how different temperatures (4, 25, 37, and 42°C) affected the ability of CN1 to remove substances; **(D)** CN1 cells in MRS broth with different bacterial quantities (4 × 10^9^, 2 × 10^9^, 4 × 10^8^, and 2 × 10^8^ CFU/mL; ZEN 10 μg/mL, pH 7.0). One-way ANOVA was used to analyze significant differences (*p < 0.05*).

The ZEN removal rate of CN1 (4 × 10^9^ CFU/mL) in MRS was tested with ZEN concentrations ranging from 2.5 to 200 μg/mL (Figure 3A). At 2.5 and 8.5 μg/mL, CN1 removed more than 50% of ZEN after 12 h. For higher concentrations (10 and 200 μg/mL), CN1 quickly removed 39.5 and 8.5% of ZEN, respectively. After 72 h of incubation, CN1 cells continued to remove more ZEN at 2.5, 8.5, and 10 μg/mL. We hypothesized that the greater the ZEN concentration and the longer the incubation period, the more likely ZEN would connect to the remaining binding sites on the outermost layer of CN1. We discovered that ZEN removal by CN1 is a quick response since CN1 cleared 56 and 53.5% of ZEN soon after contact with ZEN at levels of 2.5 and 8.5 μg/mL, respectively. At high beginning levels of ZEN (10 and 200 μg/mL), lower quantities of ZEN were removed by CN1 (39.5 and 8.5%, respectively) shortly after the inclusion of ZEN. Nonetheless, after incubating CN1 for 24 and 72 h with ZEN (10 μg/mL), the quantity of ZEN removed by the CN1 rose to roughly 43 and 69%, respectively. These findings are consistent with recent studies on the mycotoxin removal capabilities of lactic acid bacteria. Similarly, a study demonstrated that *Lactobacillus plantarum* exhibited significant efficacy in removing aflatoxin B1 and ZEN through adsorption mechanisms (Huang et al., 2018).

Moreover, a study investigated the factors influencing mycotoxin binding by lactic acid bacteria and highlighted those environmental conditions, such as pH and temperature, play a crucial role in optimizing mycotoxin removal (Nasrollahzadeh et al., 2022). After being consumed by mouth, the feed digesta passes through the animal’s gastrointestinal system in various pH states. The pH range for the pig gastrointestinal system is 6.1–6.7 for the small intestine, 4.4 for the stomach, 6.1–6.6 for the colon, and 6.0–6.4 for the caecum (AlTabbaa and Ankrah, 2016). Therefore, the efficacy of a mycotoxin-degrading agent was evaluated under different pH settings. In their experiments, optimal mycotoxin binding was observed under specific pH conditions, similar to the results observed in the current study, where CN1 showed effective ZEN removal at 2.5, 8.5, and 10 μg/mL concentrations after 24 h. The effects of pH on CN1 ability to remove ZEN were tested after 12, 24, and 72 h of treatment with ZEN at pH 3.0, 6.0, 7.0, and 8.0. The levels of ZEN eliminated by CN1 at pH 3.0 and 6.0 (10 and 17%, respectively) were much lower than those at pH 7.0 and 8.0 (39.5 and 36%, respectively). After 24 and 72 h at pH 7.0 and 8.0, the quantity of ZEN removed by CN1 rose considerably (Figure 3B).

The effects of temperature on CN1 ability to degrade ZEN found that CN1 removed over 50% of ZEN immediately at 37°C and achieved the highest removal rate of 69.01% after 72 h at this temperature (Figure 3C). The degradation rates were significantly lower at 4 and 25°C but significantly higher at 42°C. The results show that although the removal rate at 42°C was numerically higher than at 37°C, the difference was not statistically significant (*p > 0.05*), as determined by one-way ANOVA. This finding suggests that while both temperatures support effective ZEN degradation by CN1, 37°C remains the optimal condition considering both efficiency and potential thermal stress on the bacterial cells. These findings are the same as those of Hsu et al. (2018), which reported that the CK1 strain removed more ZEN at 42°C after 24 h compared to 4, 25, and 37°C. A more recent study demonstrated that another microbial strain, B73, showed optimal ZEN degradation at 30°C, with efficiency dropping significantly at higher and lower temperatures (Liu et al., 2023). Similarly, a study found that ZEN degradation by strain XZ-2 peaked at 35°C but decreased at temperatures above 40°C (Wu et al., 2024). These studies collectively suggest that optimal degradation temperatures vary significantly between microbial strains, underscoring the importance of selecting the appropriate strain and maintaining optimal environmental conditions for effective ZEN management.

### 3.7 ZEN removal by CN1 strain at different bacterial concentrations

The capacity of various concentrations of CN1 (4 × 10^9^, 2 × 10^9^, 4 × 10^8^, and 2 × 10^8^ CFU/mL) to remove ZEN in MRS broth with a ZEN concentration of 10 μg/mL over 72 h (Figure 3D). The results indicated that CN1 at higher concentrations of 4 × 10^9^ and 2 × 10^9^ CFU/mL removed 69 and 65.6% of ZEN, respectively. In contrast, lower bacterial concentrations of 4 × 10^8^ and 2 × 10^8^ CFU/mL resulted in significantly lower ZEN removal rates of 43.88 and 10.21%, respectively. These findings suggest that a critical threshold of CN1 biomass is required to achieve effective ZEN removal, as lower concentrations showed a marked reduction in detoxification efficiency. While the increase from 2 × 10^9^ to 4 × 10^9^ CFU/mL did not yield a significant difference, concentrations below 109 CFU/mL resulted in a sharp decline in performance, indicating a potential saturation point for adsorption capacity. Comparatively, a recent study investigated the impact of different bacterial concentrations on mycotoxin degradation and found a similar trend, where higher concentrations of the tested strain significantly improved the removal rates of aflatoxin B1 (Li et al., 2023). Additionally, Park et al. (2022) reported that the efficiency of ochratoxin A degradation by *Lactobacillus plantarum* increased with bacterial density, emphasizing that higher cell concentrations provide more active sites for toxin binding and enzymatic degradation.

### 3.8 ZEN removal by culture supernatant, cell walls, and bacterial contents

**Figure 4:**
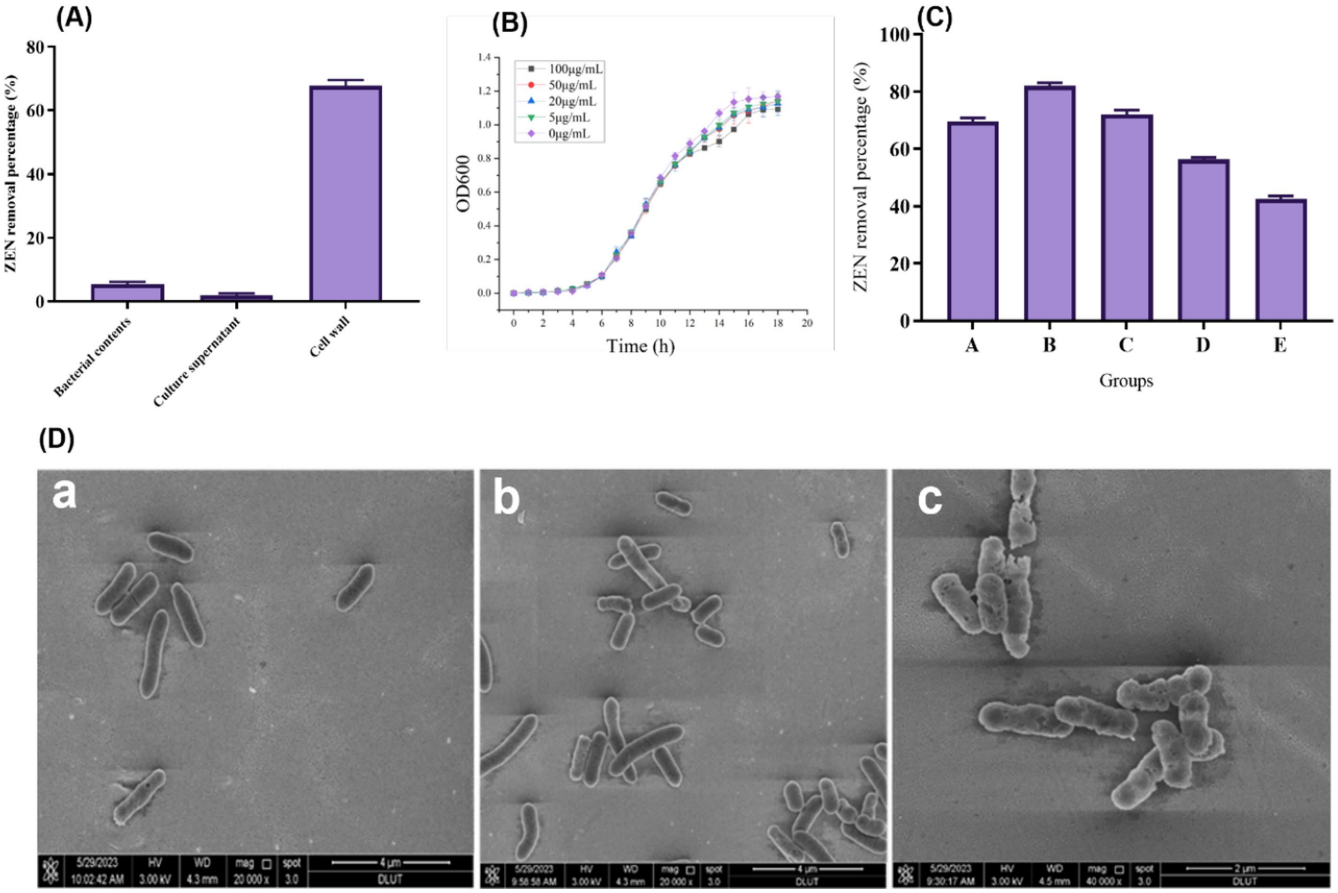
**(A)** Degradation effect of fermentation supernatant, cell wall and intracellular fluid of strain on ZEN; **(B)** Growth status of CN1 bacteria at different ZEN concentrations; **(C)** Effect of different ways to treat CN1 bacteria on ZEN removal; **(D)** Scanning Electron Microscope (SEM) images of bacterial cells at magnifications of 20,000x and 40,000x (a) Bacterial cells with no treatment; (b) Cells subjected to ZEN treatment; (c) Cells treated with both ZEN and heat. One-way ANOVA was used to analyze significant differences (*p < 0.05*).

Figure 4A shows the results of ZEN removal using the CN1 strain cell wall, culture supernatant, and intracellular contents. The cell wall effectively removes ZEN, achieving a removal rate of over 60%. In contrast, the removal rates of ZEN in the fermentation supernatant and intracellular fluid are both below 5%. This suggests that the removal of ZEN by CN1 primarily relies on the adsorption capacity of the bacterial cell wall. It can be demonstrated that this strain eliminates ZEN through enzyme secretion and cell wall action. Previous research found that the breakdown rate of *Escherichia coli* CG1061 supernatant from culture was 61.8%, but the biological degradation of intracellular isolates was only 17.6%, indicating that the active component was continuously discharged into the extracellular area. The breakdown rate was reduced to 37.5% when the cultured supernatant was treated with 1 mg/mL proteinase K but remained at 51.3% after 20 min at 100°C (Wang et al., 2019). Furthermore, research revealed that certain mycotoxins were inactivated or absorbed through the cell wall (Yiannikouris et al., 2021).

### 3.9 Characterization of ZEN adsorption mechanism

After exposing the bacterial strain to ZEN, an SEM examination was conducted to investigate their shape and fundamental makeup. The alterations in bacterial cells following ZEN treatment are depicted in Figure 4D. When compared to the control sample, SEM pictures acquired at levels of magnification of 20,000x and 40,000x demonstrated visible damage to the treated cells (a). The strains exhibited elongation and thinning when cultured in an MRS medium, warranting further investigation. Moreover, SEM examination after the incubation of the two treatments in an MRS medium with ZEN revealed minimal differences in bacterial morphology (b). However, after heat and ZEN treatment, increased cell damage was observed (c). These observed modifications may elucidate the diverse adsorption percentages achieved after ZEN adsorption and suggest an alternative cellular wall structure. Additionally, SEM examination revealed intercellular connections among bacterial strains throughout the adsorption process. Furthermore, SEM revealed elusive variations in bacterial morphology after incubation in LAPTg and MRS medium, consistent with our study’s findings. Specifically, *Bacillus* strains exhibited a more elongated and thin morphology when cultivated in LAPTg compared to MRS (Adunphatcharaphon et al., 2021; Murtaza et al., 2023a). This intriguing characteristic of the strain warrants further investigation in future studies. Importantly, the results suggest that these treatments had no appreciable effect on the ratio of chemical components on the bacterial cell surfaces. The glycopolymers and proteins that make up the cell wall of typical *Lactobacillus* are intricately arranged. Proteins, polysaccharides, and teichoic acids are used to adorn the thick peptidoglycans that encase the cytoplasmic membranes (Chapot-Chartier and Kulakauskas, 2014). The pattern and structure of bacterial cell surfaces were changed as a result of alterations in the sugars and amino acids found in glycopolymer or protein structures (Stefanović et al., 2021). This shows that the *Lactobacillus* cell wall’s distinctive structure and content are key to the organism’s ability to detoxify ZEN.

### 3.10 Bacterial tolerance to ZEN

Since ZEN is toxic to cells, its presence can impact the growth of microbial strains. Therefore, high tolerance to ZEN is essential for practical applications. Our results indicate that the growth of CN1 was only slightly affected at a ZEN concentration of 100 μg/mL, as shown in Figure 4B. This suggests that CN1 exhibits strong tolerance to ZEN. These findings are consistent with previous studies that have highlighted the resilience of certain lactic acid bacteria to mycotoxins. For instance, a study demonstrated that some *Lactobacillus* strains could tolerate and even reduce ZEN concentrations in contaminated media through adsorption mechanisms (Topcu et al., 2010). Similarly, research found that *Lactobacillus rhamnosus* strains were capable of binding and sequestering ZEN, thereby reducing its bioavailability and toxicity (Fuchs et al., 2002). The ability of CN1 to withstand high levels of ZEN and maintain growth is particularly advantageous for applications in food and feed safety. Moreover, the strong tolerance of CN1 to ZEN supports its potential use in fermentation processes aimed at detoxifying contaminated feedstocks, thereby improving their safety and nutritional quality.

### 3.11 Effect of different treatments on ZEN removal by CN1 strain

To investigate the adsorption effect of CN1, various treatments were applied to the bacteria, as illustrated in Figure 4C. The untreated group (Group A) demonstrated an adsorption rate of over 65%. Heat treatments significantly enhanced adsorption capabilities: Group B, subjected to autoclaving, achieved an adsorption rate of 82.9%, while Group C, treated in a 100°C water bath, exhibited a rate of 73.3%. In contrast, the adsorption efficiency decreased following acid and alkali treatments. Specifically, Group D, treated with acid, showed an adsorption rate of 56.2%, and Group E, treated with alkali, had a rate of 43.5%. These findings align with previous research that has shown the impact of different treatments on the adsorption abilities of lactic acid bacteria. For instance, studies have demonstrated that heat treatment can expose more binding sites on the bacterial cell wall, enhancing the adsorption of toxins such as ZEN. Our study reported similar improvements in adsorption rates following heat treatment of *Lactobacillus* strains, suggesting that thermal processes can denature cell wall proteins, thereby increasing their binding capacity for mycotoxins (Murtaza et al., 2023a). Conversely, the reduction in adsorption following acid and alkali treatments can be attributed to the alteration or degradation of cell wall components, which play a crucial role in toxin binding. Research found that extreme pH conditions could negatively affect the structural integrity of bacterial cell walls, thereby diminishing their adsorption efficiency (Niderkorn et al., 2006).

### 3.12 Stability of ZEN adsorption by CN1 strain

**Figure 5:**
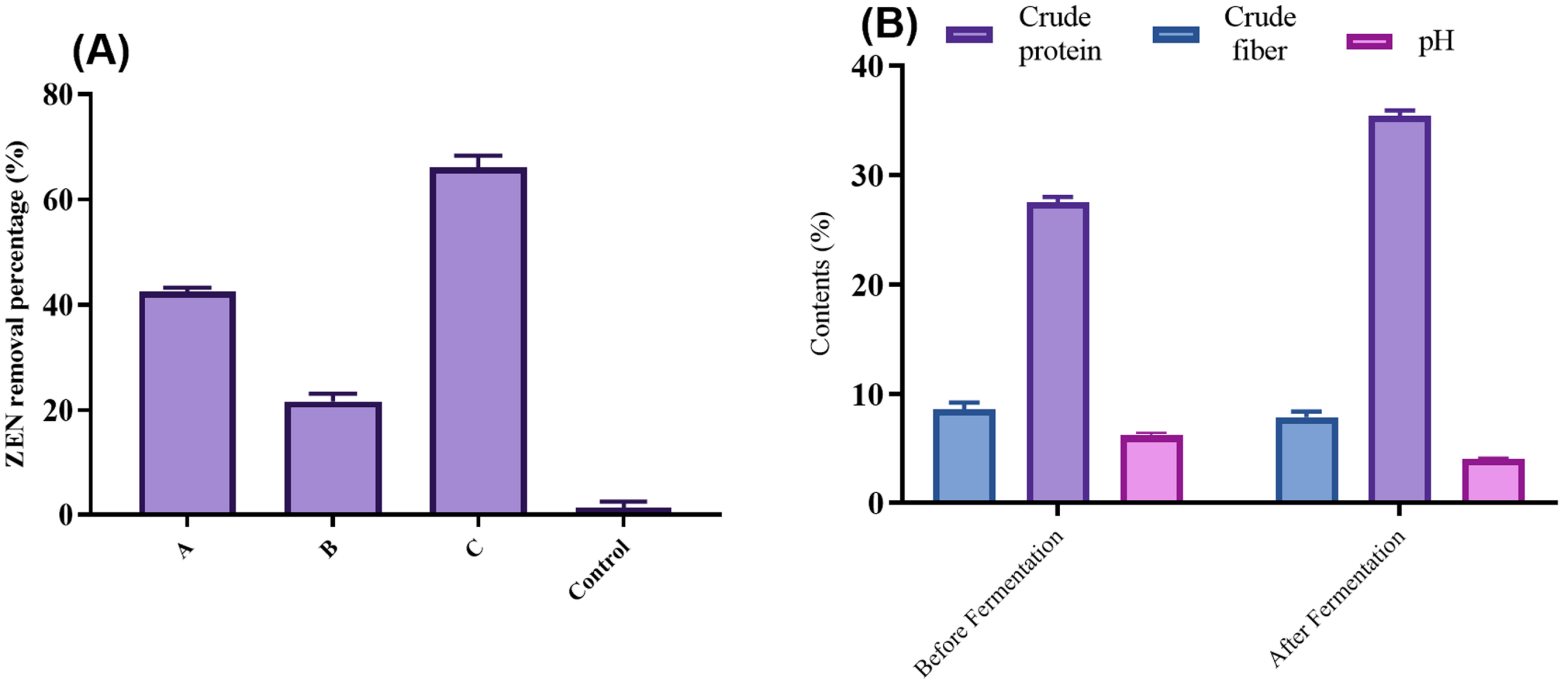
**(A)** Desorption rate of cell wall ZEN complex after different treatments; **(B)** Changes of crude fibers, crude protein, and pH content before and after DDGS fermentation. One-way ANOVA was used to analyze significant differences (*p < 0.05*).

In this study, CN1 removed ZEN through physical adsorption by its cell wall. However, under various external conditions, ZEN adsorbed on the cell wall may desorb back into the system. Therefore, the stability of the ZEN-cell wall conjugate was investigated. After treating the ZEN-adsorbed bacteria with heating, shaking, and organic solvents (Figure 5A), some ZEN was detected in the system. The desorption effect was most pronounced with organic solvent washing, which had a desorption rate as high as 65%. Heat treatment resulted in the separation of 43.4% of ZEN from the complex, while the shaking method had the least effect, with a desorption rate of only 22.1%.

These results indicate that the adsorption of ZEN by the microbial cell wall is reversible. Consequently, caution is required when using this method in production, and attention must be paid to the storage conditions of subsequent products to prevent the re-release of ZEN. This reversibility aligns with findings from other studies. A study found that the binding of mycotoxins to yeast cell walls could be influenced by environmental conditions, potentially leading to desorption under certain circumstances (Kihal et al., 2022). Additionally, Johnson (1990) demonstrated that the stability of toxin adsorption could be affected by pH and temperature changes, reinforcing the need for careful consideration of storage and handling conditions. In practical applications, these findings underscore the importance of optimizing both the adsorption process and subsequent storage conditions to maintain the efficacy of mycotoxin removal.

### 3.13 Determination of nutritional components after fermentation

As illustrated in Figure 5B, fermentation significantly altered the nutrient profile of DDGS. Post-fermentation analysis revealed a 0.79% decrease in crude fiber content, enhancing digestibility for livestock and poultry. High cellulose levels in DDGS typically exceed feed standards, so reducing cellulose content is beneficial for increasing its use in the breeding industry. This reduction aligns with findings that lower fiber content improves nutrient absorption and feed efficiency in livestock (Jha and Berrocoso, 2015).

Conversely, crude protein content increased by 7.65% after fermentation. This rise is primarily due to the proliferation of CN1 during fermentation, which produces bacterial and exocrine proteins while consuming energy. Increased microbial protein synthesis during fermentation is well documented and improves the nutritional quality of feed (Olukomaiya et al., 2020). Additionally, the pH of DDGS dropped significantly from 6.5 to 4.2 post-fermentation. This reduction is attributed to the anaerobic fermentation of CN1, creating an acidic environment that inhibits the growth of harmful bacteria and molds, thereby extending shelf life. Notably, after 180 days, no mold growth was observed under high moisture conditions, underscoring the effectiveness of fermentation in prolonging feed stability. Finally, the fermented DDGS exhibited a more pronounced lactic acid taste, potentially enhancing palatability and food intake for animals. This improvement in taste could lead to higher feed consumption, although further verification is needed. Enhanced palatability through fermentation has been supported by other research, suggesting that lactic acid bacteria can improve feed flavor and acceptance (Bintsis, 2018).

### 3.14 ZEN determination after fermentation

As shown in Table 1, ZEN in the sample was extracted and tested with a ZEN ELISA detection kit. The recovery rate of ZEN reached 94.7%, confirming the feasibility of the extraction and detection method. For DDGS feed products fermented by CN1, the ZEN content was measured post-fermentation using the same kit, revealing a removal rate of 75.7%. This indicates that the CN1 fermentation method is highly effective at removing ZEN, surpassing the capabilities of other strains. Additionally, the CN1 fermentation process enhances the degradation effect, optimizing the reduction of ZEN in DDGS feed products. Previous studies have extensively explored methods for removing mycotoxins, including ZEN, from agricultural products and feed. Many of these studies have focused on utilizing microorganisms like lactic acid bacteria (LAB) due to their ability to degrade mycotoxins through various mechanisms. For instance, the study by Li et al. (2020) demonstrated the efficient removal of ZEN from contaminated grains using LAB strains, with removal rates ranging from 40% to over 90% depending on the strain and conditions. Similarly, the work highlighted the potential of LAB in reducing ZEN levels in feed materials, achieving removal rates exceeding in some cases (Chen et al., 2018). These findings align with the current study’s results, which show a remarkable removal rate of 75.7% using CN1 fermentation on DDGS feed. Furthermore, studies investigating the stability and reversibility of ZEN adsorption on microbial cell walls have emphasized the importance of understanding desorption processes. Research explored the desorption behavior of ZEN from LAB cell walls under different conditions, corroborating the current study findings regarding the reversibility of ZEN adsorption. The high recovery rate (94.7%) of ZEN extraction using the method in this study aligns with previous research demonstrating the reliability and accuracy of ELISA-based detection kits for mycotoxins in agricultural samples.

**Table 1:** ZEN recovery rate verification results.

ZEN concentration (μg/mg)	Detected concentration (μg/mg)	Recovery rate (%)
2	0.85	85
5	4.70	94.50
10	9.50	95.50

## 4 Conclusion

This study presents an innovative approach to mitigating ZEN contamination in DDGS through probiotic fermentation using CN1. The results demonstrate that CN1 can significantly reduce ZEN levels by up to 75.6%, primarily through adsorption to the bacterial cell wall, with heat treatments further enhancing adsorption efficiency to 82.9%. This method not only surpasses the effectiveness of traditional physical adsorption techniques, which often compromise feed quality, but also improves the nutritional profile of DDGS by increasing crude protein content by 7.16% and reducing crude fiber by 0.65%. Scanning electron microscopy revealed structural changes in CN1 cells under stress conditions, contributing to their enhanced adsorption capacity. The dual benefit of detoxification and nutritional enhancement offers a novel, practical solution for improving feed safety and economic value. This approach holds significant potential for large-scale industrial application, particularly in regions with high mycotoxin contamination, and could set a new standard in sustainable agricultural practices. Future research may explore its application to other mycotoxins or feed types, optimizing the process for diverse conditions and combining it with other strategies for even greater efficacy.

## Data Availability

The original contributions presented in the study are included in the article/supplementary material, further inquiries can be directed to the corresponding authors.
